# Lazy Resampling: Fast and information preserving preprocessing for deep learning

**DOI:** 10.1016/j.cmpb.2024.108422

**Published:** 2024-12

**Authors:** Benjamin Murray, Richard Brown, Pengcheng Ma, Eric Kerfoot, Daguang Xu, Andrew Feng, Jorge Cardoso, Sebastien Ourselin, Marc Modat

**Affiliations:** a School of Biomedical Engineering & Imaging Sciences, King’s College London, London, UK; b NVIDIA Santa Clara, CA, USA

**Keywords:** Deep learning, Medical images, Preprocessing, Lazy resampling

## Abstract

**Background and Objective:**

Preprocessing of data is a vital step for almost all deep learning workflows. In computer vision, manipulation of data intensity and spatial properties can improve network stability and can provide an important source of generalisation for deep neural networks. Models are frequently trained with preprocessing pipelines composed of many stages, but these pipelines come with a drawback; each stage that resamples the data costs time, degrades image quality, and adds bias to the output. Long pipelines can also be complex to design, especially in medical imaging, where cropping data early can cause significant artifacts.

**Methods:**

We present Lazy Resampling, a software that rephrases spatial preprocessing operations as a graphics pipeline. Rather than each transform individually modifying the data, the transforms generate transform descriptions that are composited together into a single resample operation wherever possible. This reduces pipeline execution time and, most importantly, limits signal degradation. It enables simpler pipeline design as crops and other operations become non-destructive. Lazy Resampling is designed in such a way that it provides the maximum benefit to users without requiring them to understand the underlying concepts or change the way that they build pipelines.

**Results:**

We evaluate Lazy Resampling by comparing traditional pipelines and the corresponding lazy resampling pipeline for the following tasks on Medical Segmentation Decathlon datasets. We demonstrate lower information loss in lazy pipelines vs. traditional pipelines. We demonstrate that Lazy Resampling can avoid catastrophic loss of semantic segmentation label accuracy occurring in traditional pipelines when passing labels through a pipeline and then back through the inverted pipeline. Finally, we demonstrate statistically significant improvements when training UNets for semantic segmentation.

**Conclusion:**

Lazy Resampling reduces the loss of information that occurs when running processing pipelines that traditionally have multiple resampling steps and enables researchers to build simpler pipelines by making operations such as rotation and cropping effectively non-destructive. It makes it possible to invert labels back through a pipeline without catastrophic loss of accuracy.

A reference implementation for Lazy Resampling can be found at https://github.com/KCL-BMEIS/LazyResampling. Lazy Resampling is being implemented as a core feature in MONAI, an open source python-based deep learning library for medical imaging, with a roadmap for a full integration.

## Highlights

• Lazy Resampling can improve the performance of deep neural networks.
• Lazy Resampling mitigates the information loss typical in preprocessing pipelines.
• Lazy Resampling’s non-destructive operations enable simpler pipelines.
• Lazy Resampling can preserve label accuracy, particularly when mapping labels back to source data.
• Lazy Resampling is designed to be simple to use, is integrated into MONAI, and is enabled with a single flag.

## Graphical abstract

**Figure:**
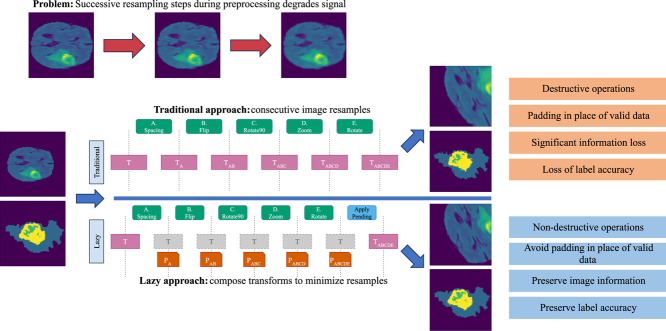

## 1 Introduction

Deep neural networks (DNNs) have transformed medical image processing in recent years, providing new state of the art in automating many computer vision tasks, including semantic segmentation [1]. Deep learning models require considerable tuning to function well, with data preprocessing pipelines being an important part of model tuning. Such pipelines serve both to prepare data to be suitable for ingestion by a neural network, and to provide data augmentation that encourages networks to learn a more generalised representation of a task, especially in circumstances where training data is limited, as is often the case for medical imaging applications of deep learning.

Processing pipelines are typically composed of a sequence of transforms. Each transform has a specific operation that is performed on the data, such as loading the data, reordering dimensions to follow conventions for a given deep learning library, spatially modifying the data, cropping a subset of the data, or adding noise to the data. Each of these operations is carried out by a given transform without regard for prior or subsequent transforms, and can be considered destructive, since the data passed to a transform is either modified in place or a new, modified copy of the data is returned after the transform’s execution.

Depending on the pipeline, special care must be taken by the pipeline designer in order to avoid situations where part of the data is effectively destroyed by an early transform and thus is unavailable for a subsequent transform despite still being required. This is often the case with memory-intensive volumetric medical images, which use methods such as patching [2] to reduce the amount of memory required to train a given network. Such pipelines must be designed with sufficient padding in mind to avoid cropping of important data, which can be overlooked by users and yield sub-optimal results. Padding the data also increases the memory requirements and time taken to execute preprocessing.

Even if pipelines are designed to avoid unwanted destruction of data by spatial or cropping transforms, the concatenation of many resampling operations themselves can result in excessive information loss. This is particularly relevant in medical imaging, where images often contain small or subtle but relevant details that lack much contrast with their surroundings.

In this paper, we present a lazy resampling architecture for data pipelines which merges discrete image resampling operations within a pipeline wherever it is possible to do so. It converts sequences of individual affine or grid-based transforms into composite operations that produce equivalent outputs from a given input. Our Lazy Resampling method improves the performance, simplicity, and image quality of the preprocessing pipeline, with the following benefits:

*Performance:* Improved preprocessing pipeline performance reduces the time taken to perform hyperparameter searches [3] and the time taken to train tuned model architectures. This allows finer-grained hyperparameter searches or searches with more representative preprocessing pipelines. It also improves the energy budget required to train new models.

*Simplicity:* By making spatial and preprocessing operations non-destructive, Lazy Resampling avoids the additional complexity that must otherwise be designed into pipelines to avoid unnecessary data loss.

*Information preservation:* Lazy Resampling mitigates the loss of information in a given preprocessing pipeline. This is because a typical lazy resampling pipeline requires fewer resampling operations than corresponding traditional pipelines, inducing less signal degradation from resampling operations.

Critically, from a user perspective, our method is implemented in such a way that users do not need to learn new ways of defining preprocessing pipelines; our open-source implementation uses all the same conventions as other preprocessing libraries for deep learning and is effectively identical from an application programming interface (API) perspective.

In this paper we present Lazy Resampling, the concepts behind it, and our open-source design and implementation. We show how it improves on existing preprocessing libraries through use of computational geometry at the heart of its design, and the flexibility, simplicity and information preservation that occurs as a result. We show how we innovate from an engineering standpoint by allowing users to take advantage of the power of computational geometry based transforms without them needing to understand any of the mechanisms that underlie its implementation. These contributions serve together to overcome the limitations of existing technologies. We present experiments demonstrating that Lazy Resampling is not only faster, but is able to improve network performance when applied to the training of semantic segmentation networks.

## 2 Material and methods

### 2.1 Related work

#### 2.1.1 Semantic segmentation

Semantic segmentation is the process of determining semantic classification labels for each element of a data sample. For imaging data, this means a label per pixel or voxel of the data. Semantic segmentation is heavily used in clinical applications where an organ or pathology of interest must be highlighted within the data sample. Semantic segmentation by hand is extremely time-consuming, particularly when dealing with voxel data and, in medical imaging, must typically be done by qualified radiologists, whose time is always limited. The goal of automating semantic segmentation in a clinical setting is to free up the time of radiologists by having DNNs perform the necessary semantic segmentations. Applications for semantic segmentation include identification of and surgery planning for pathologies such as Glioma [4].

#### 2.1.2 Processing pipeline design

All deep learning libraries have preprocessing functionality. PyTorch [5], a popular deep learning framework, provides *Datasets*, *DataLoaders* and *Compose* classes for this purpose.

*Compose* is a top-level transform containing the sequence of transforms to be performed and knows how to iterate over them when called. *DataSet* is passed the compose instance and calls it with the appropriate data sample before returning it. *DataLoader* is passed the dataset instance and iterates over it, performing operations such as shuffling the dataset order and batching samples. PyTorch does not have a native transform library, but *TorchVision*^1^ is its de-facto library. However, many popular transform libraries have been written, and libraries tend to be specialised to given domains. There are a number of popular medical imaging-focused libraries, such as *torchIO* [6] and *batchgenerators*.^2^ It is common for users to mix transforms from different libraries in a preprocessing pipeline.

All transform libraries work in essentially the same way. Transforms, given user-defined parameters and an optional random number generator or seed value for a random number generator, take a tensor, perform the specified transformation which may be deterministic or stochastic depending on the transform, and return the transformed tensor instance. Depending on the transform, this operation can be a lossless tensor operation or a lossy resampling operation. For example, flip operations are always lossless as they simply permute the values in the tensor. Rotations outside of 90-degree angles are always lossy, as they involve interpolation operations that generate new pixel values.

#### 2.1.3 Lazy evaluation

Lazy evaluation is a common concept in computer science and software engineering, and refers to the act of deferring some expensive task until it becomes necessary to carry it out. The ‘lazy’ aspect of Lazy Resampling is that, for any given transform that is designed for lazy operation, we attempt to avoid the expensive resampling operation, preferring to collate a number of operations together before we perform the resampling step.

#### 2.1.4 Homogeneous matrices and computer graphics

The fields of computer graphics and robotics have long made use of techniques from linear algebra to generate efficient, composable representations of operations on Euclidean space. Homogeneous matrices are able to represent all linear transformations in Euclidean space in a manner that is composable and, for linear transforms that have a non-zero determinant, invertible. These have become the de-facto method for describing operations in rendering and simulating virtualised geometries, and performing animations of and within virtualised geometries [7]. They can be used in a hierarchic fashion to control the position and orientation of body parts of animated figures or robots, where each part’s position is described relative to a parent part. The orientation of a part in the world can be determined by multiplying the matrices representing the transforms of the parent parts from the root to the part in question.

#### 2.1.5 Composed spatial and grid transformations in computer vision

In medical computer vision, there are a number of scenarios in which composition of homogeneous matrices is used. For example, image registration tools using iterative methods must perform many resampling operations on grids of vectors. In deep learning preprocessing libraries, this concept can also be found, but to a limited extent relative to our implementation. Some libraries have *Affine* transforms that allow a number of spatial operations to happen before a resample. These tend to have a fixed set of operations, fixed ordering, and limited configurability with respect to randomness, and undergo a resample even if they are not the final transform in a pipeline. *rising*^3^ is the preprocessing library whose functionality comes closest to a full Lazy Resampling implementation, but requires that all transforms that have composable spatial transforms to be wrapped in a *StackedAffine* transform instance. Furthermore, our Lazy Resampling implementation is engineered to provide the maximum benefit to a typical user without requiring them to modify their pipelines or even think about their pipelines in terms of affine transforms.

### 2.2 Problem statements

This section delves further into issues that arise when using non-lazy preprocessing pipelines.

#### 2.2.1 Resampling noise reduces signal

**Fig. 1:**
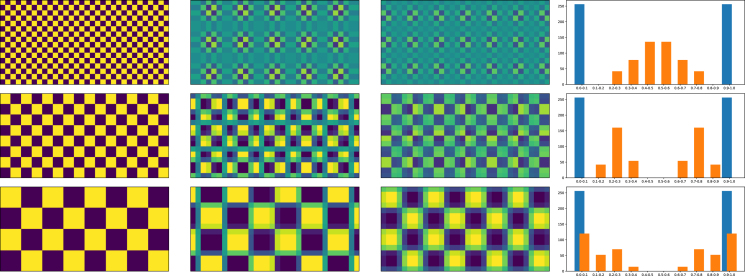
Moire pattern artifacts as a result of applying a scale factor of 6/5 and inverting it by applying a scale factor of 5/6. The first column contains checkerboard patterns of 1 × 1, 2 × 2 and 4 × 4 pixels, respectively. The second column shows the images after the first scaling operation. The third column shows the image after it has been scaled and then inverted. The right-most column shows histograms, blue for the original image and orange for the image that has been scaled and inverted.

Noise introduced by successive interpolation layers can significantly degrade the signal required for the network to train. While noise can certainly be beneficial for augmentation during training, interpolation noise from geometric transforms can add significant bias. Fig. 1 shows an example of moire artifacts on chequerboard data introduced by linear interpolation using the linear interpolation technique, the interpolation mode typically used when interpolating image data in a preprocessing pipeline. Although the chequerboard maximises the effect of the moire patterning, we can see that small features can be substantially altered and the overall data distribution significantly changed by interpolation.

A further issue with resampling noise is that, typically, image data is linearly interpolated but segmentation labels are nearest neighbour interpolated. A deep pipeline can cause severe degradation of the labels that is more confounding to the network than the loss of image quality, as is highlighted by the experiment performed in Section 3.2.

#### 2.2.2 Patches in traditional pipelines

All resampling operations can be thought of as destructive. Any data that is not within the destination region of interest being resampled is effectively lost. Further transformations can then result in a viewport that is within the range of the original data, but outside of the remaining cropped data. A simple illustration is to translate an image by a given number of units and then translate it back by the same number, the resulting sample will contain padding instead of values existing in the original image.

Lazy Resampling addresses this issue, as, until a resample operation occurs, the information is not destroyed. The patch sampling operation (taking a region of a sample) is effectively a resized viewport with a translation and so the patch is only effectively taken at the point the resample is taken. The top row of Fig. 5 demonstrates the difference between traditional and lazy pipelines that perform a patch-based sample operation up-front.

#### 2.2.3 Inversion

Many spatial operations have the ability to be undone mathematically. For example, a rotation of 45° can be undone by a rotation of −45°. The latter rotation is the *inverse* of the former. When represented as a homogeneous matrix, the inverse of a transform can be obtained by calculating the inverse of the matrix via standard linear algebra. Note that not all transforms have inverses, but, provided all of the transforms are invertible, the composed matrix representing those transforms is also invertible.

The ability to invert a set of applied preprocessing transforms allows users to spatially match up network output with the untransformed data samples. This is particularly useful for datasets that must be learned in patches due to data sample size, as the patches can be superimposed over the corresponding region of the source data for direct comparison. This doubles the number of resampling steps that must occur however, further exacerbating image degradation. This is also true for Lazy Resampling, but only involves as many additional resamples on the inversion pass as are required for the forward pass.

### 2.3 Design and implementation

Lazy Resampling works by building up a list of pending transform operations rather than executing a transform’s operation on the data at the point that the transform is called. It defers the execution of the list of pending transforms until their execution is required in order for the next transform to operate correctly. Then, rather than carrying out each transform individually, it composites the specified operations together in order to generate a single resample operation for all of the pending operations.

In order to do this, we separate the description of a spatial transform from its execution. Each transform instead provides a description of the pending transform to be performed that can be composed with other pending transforms at the point of resampling. We do this by wrapping pytorch tensors in a *MetaTensor* class that adds metadata to the tensor so that pending and applied operations can be tracked with the tensor.

**Fig. 2:**
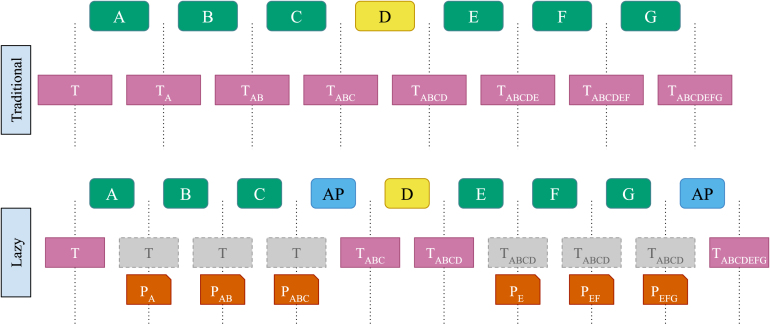
Illustration of resampling operations in traditional and lazy pipelines. In the traditional pipeline, tensors (labelled T) are modified with each transform that is executed, with the applied operations being in subscript. Lazy execution, by contrast, avoids making copies of tensors where possible by instead building a pending list of transforms (labelled P). The pending list only gets applied and a new tensor generated at the point that it is executed by an ApplyPending (labelled AP in the figure.) transform. Greyed-out tensors indicate that executing the transform returns the same tensor. The first ApplyPending applies $P_{ABC}$, resulting in a new tensor $T_{ABC}$. Transform D does not execute lazily, so its output is the tensor $T_{ABCD}$. The second ApplyPending transform applies $P_{EFG}$ to $T_{ABCD}$ resulting in the final tensor $T_{ABCDEFG}$.

The pending transforms are gradually accumulated as the data progresses through the pipeline until it becomes necessary to resample. A resample can be triggered by any of the following operations: reaching the end of the pipeline, encountering transforms that cannot operate lazily, or encountering transforms with incompatible parameters (e.g. a change in sample mode or data type). Fig. 2 illustrates this process in traditional vs. lazy resampling pipelines.

#### 2.3.1 Lazy transform pending description

A pending transform contains a description of the spatial transform and associated metadata:

• operation The spatial operation to be performed
• input_shape The extents (pixel count in each spatial dimension) of the input data sample in pixels
• input_dtype The datatype of the input data sample
• output_shape The extents of the output data sample in pixels
• output_dtype The datatype of the output data sample
• interpolation_mode The interpolation mode expected for this operation when it is applied
• padding_mode The padding mode expected for this operation when it is applied

operation is either a homogeneous transform (N∗N elements) or a grid of vectors ($\prod_{i=1}^{N}d_{i}*N$ elements) where N is the number of spatial dimensions +1.

#### 2.3.2 Compose

*PyTorch* provides a standard *Compose* class that supports the execution of transforms that take a single argument (typically a tensor or a dictionary) and return a single argument. Other libraries, such as MONAI [8], provide modified *Compose* implementations that natively handle concepts such as multi-sampling transforms. In order for Lazy Resampling to have the maximum possible compatibility with existing *Compose* implementations, we introduce the concept of *compilation* to transform pipelines. We introduce a new mechanism, the *PipelineCompiler* class, that takes a list of transforms and transforms them into a modified, nested transform sequence that can be executed in a vanilla *Compose* implementation. We also provide a lazy resampling aware implementation of *Compose* that uses the *pipeline compiler* internally.

#### 2.3.3 Pipeline compiler

The *pipeline compiler* works much like a compiler for computer languages. It takes a list of transforms, and modifies that list so that it can be executed appropriately. For execution of lazy transforms, this involves the following operations:

• Insertion of *ApplyPending* transforms where appropriate.
• Insertion of *MultiSampler* transforms around lazy transforms that generate multiple samples.

#### 2.3.4 ApplyPending

The *ApplyPending* transform carries any pending transforms and returns a tensor with the applied transforms. It is inserted by the *PipelineCompiler* when a lazy transform executing lazily (*lazy == True)* is followed by a non-lazy transform. It is also inserted after any lazy transforms that are not being executed lazily (*lazy == False*).

#### 2.3.5 MultiSampler

**Fig. 3:**
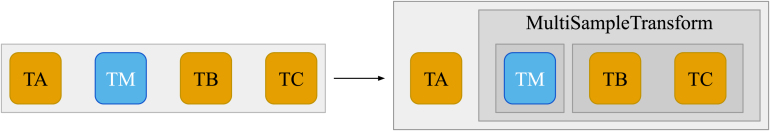
Illustration of the compilation of the pipeline on the left by the *PipelineCompiler*. Transform *TM* is a transform that is capable of multi-sampling. The pipeline compiler creates a *MultiSampler* transform, which itself is a container. *TM* is the first transform in the container and will be called in an iterative fashion until all samples have been performed. *TB* and *TC* are downstream of *TM* and so they are also added to the *MultiSampler* and will be executed once per sample.

As traditional patch-based sampling is a destructive operation, the tensors that result from multi-sample patching operations tend to use less memory than the full data sample from which they are taken. However, a large number of samples can still create memory pressure if they are generated at the same time. The *PipelineCompiler* wraps multi-sample lazy transforms in a *MultiSampler* object. This executes the multi-sample transform iteratively, executing the downstream transforms in a depth-first fashion, once per sample. This allows the preprocessing pipeline to execute in a manner that uses constant memory regardless of the number of samples being taken. An example of the use of the *MultiSampler* is shown in Fig. 3.

#### 2.3.6 Transform execution

Once the transform pipeline is compiled, the resulting transform is executed as a sequence of transforms. As with standard *Compose* functionality, calling a transform that contains other transforms will execute those transforms before returning.

#### 2.3.7 Mixing lazy and non-lazy transforms

Lazy Resampling is designed to be as close as possible to the existing *PyTorch* preprocessing pipeline design. In the implementation of Lazy Resampling integrated into MONAI, the user is able to set a flag to enable lazy resampling and immediately gain the benefits of eliminating at least some intermediate resampling operations. Lazy-aware transforms can be mixed with non-lazy transforms, although these will cause intermediate resamples to occur should they be called with pending transforms.

#### 2.3.8 Lazy transforms

Lazy transforms inherit an interface *LazyAttr* indicating that they are able to function lazily. All lazy transforms are required to have a *lazy* flag that is set during initialisation but can optionally be overridden at call time.

When executing lazily, lazy transforms return a description of the operation that will subsequently be performed when pending transforms are next applied. In the case that multiple lazy transforms are in sequence, a pending list of lazy transform descriptions is concatenated, as previously discussed.

#### 2.3.9 Applying pending transforms

When it is time to apply pending transforms, this is done as follows. Given a list of pending transforms:

• Create an identity composed_transform
• For each pending_transform in the pending transform list
  – If the pending_transform is compatible with the composed_transform
    * Add the pending_transform to the composed_transform
  – Otherwise
    * Apply the composed_transform
    * Set the composed_transform to be the pending_transform
• Apply the remaining composed_transform

This may occur at multiple points in the pipeline if lazy and non-lazy transforms are interleaved, as illustrated in Fig. 2.

#### 2.3.10 Universal resampling

Lazy spatial and cropping transforms are modified to decouple the description of an operation from its execution. All lazy transforms define their operations in terms of a homogeneous matrix and appropriate metadata and defer the execution of those operations to a separate resampling function. This universal resampling function takes a composed sequence of transforms and metadata and applies them to the input data when it is necessary to do so.

A potential drawback with this approach is that spatial transforms can take advantage of the characteristics of the transforms that they define. Flip and 90-degree rotations, for example, can be defined as inexpensive tensor operations with the additional benefit of being exact and thus lossless. Resamples based on homogeneous matrix transforms are relatively expensive compared to tensor operations and generally lossy as they require out-of-sample interpolation operations.

*Example:* Consider a pipeline that carries out the following sequence of operations - *Rotate(45), Zoom(1.25), Rotate(22), Rotate(−22), Zoom(0.8), Rotate(−45)*.

The transformation matrix resulting from this sequence of transforms is effectively an identity matrix. Technically, no transformation should need to be carried out, but this can only be determined by a careful analysis of the transform.

**Fig. 4:**
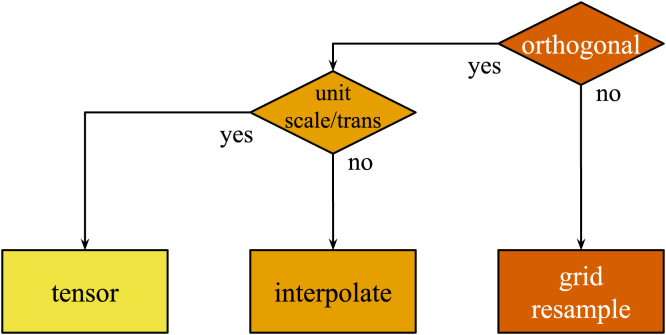
Illustration of the universal resampling logic. If the matrix does not represent an orthogonal orientation (rotations of either 0 or multiples of 90 degrees), a full resample must be performed. If the transform is orthogonal, now check whether it is scaled or translated by non-integer amounts. If it is, an interpolation operation must be performed. Otherwise, the operation can be performed directly on the data, typically via methods on the tensor.

We resolve this issue by designing the universal resampling operation to analyse and exploit the composed transform and make use of the most accurate, least expensive operation that can be performed given the transform characteristics. This is shown in Fig. 4.

## 3 Results

To demonstrate the difference between the traditionally implemented processing pipelines and the proposed lazy approach, we have designed a set of experiments aiming to quantify the image degradation induced by each approach. We also quantify the difference when training a deep neural network (DNN) on a semantic segmentation task. We use transforms from MONAI [8] to create the traditional preprocessing pipelines.

Experiments are carried out on two datasets from the Medical Segmentation Decathlon dataset [9].

The first dataset selected is the Medical Segmentation Decathlon Brain Tumour task. It is composed of 484 labelled (and 266 unlabelled) samples of four MRI sequences (FLAIR, T1w, T1gd, and T2w) resampled to 240 × 240 × 155 ($1\;{\mathrm{mm}}^{3}$) voxel volumes. The training volumes have three semantic segmentation label classes, gadolinium-enhancing tumour, peritumoural edematous/invaded tissue and necrotic tumour core [4], [9]. Foreground labels range between 0.0816% and 3.57% of a volume.

The second dataset selected is the Medical Segmentation Decathlon Hippocampus task. It is composed of 263 labelled (and 131 unlabelled) samples of T1-weighted MPRAGE MRI. The smallest volume is 34 × 53 × 24 voxels and the largest volume is 41 × 48 × 47 voxels. Foreground labels are the anterior and posterior of the hippocampus and range between 3.76% and 7.48% of a volume.

### 3.1 Forward pass comparison

In this initial experiment, we assess the degradation that comes with complex transform pipelines. We look at pipelines for whole images and patch-based sampling applied to the Brain Tumour dataset:

Whole volume pipeline: Spacing →Flip →Rotate90 →Zoom →Rotate
Patch pipeline: Patch →Spacing →Flip →Rotate90 →Zoom →Rotate

Table 1 presents the required time for each pipeline and for each approach: traditional and lazy. It also presents the decrease in Shannon entropy [10] with data binned into 256 bins when comparing the input image and the output images, as a way to quantify the information loss occurring as part of the processing steps. All results are averaged over all 484 training volumes of the Brain Tumour dataset.

It can be observed that overall, and starting from the same input data, the traditional approach produces higher entropy images than its equivalent lazy counterpart. A higher value indicates a higher randomness state and, thus, less structured data. This effect is induced by the additional resampling steps in the traditional approach that decrease the original contrast. A dependent T test performed between the means of the corresponding volumes in the traditional and lazy outputs indicates a statistically significant improvement in information retention (p-value <1e−9) for both the whole volume pipeline and the patch-based pipeline.

**Table 1:** Performance comparison and entropy measures for whole volume and patch-based traditional and lazy pipelines, carried out on samples from the Medical Segmentation Decathlon Brain Tumour dataset.

Pipeline	Time (s)		Entropy	
	Traditional	Lazy	Traditional	Lazy
Whole volume	0.577	0.339	2.279±0.594	2.158±0.571
Patch-based	0.416	0.253	3.748±0.897	3.631±0.945

### 3.2 Inverse transform comparison

**Fig. 5:**
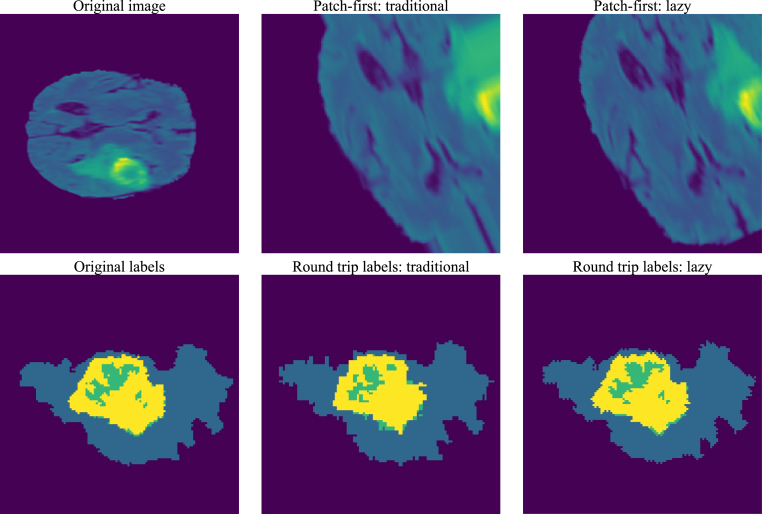
The top row shows the effects of patch-first resampling causing visual artifacts where data has been destructively cropped during the patching process and is thus not available for subsequent operations. The artifacts in the central image arise due to the ‘border’ padding policy filling in for pixels that have been destroyed by the earlier crop. Lazy Resampling performs a non-destructive crop and so is able to recover data after rotate has been applied. The bottom row shows the resulting segmentation labels following a round trip forward/inverse pass, relative to the original labels.

This experiment demonstrates the difference in preserved semantic segmentation label accuracy when round-tripping a segmentation for traditional and lazy pipelines. Again, this experiment is carried out on the Brain Tumour dataset. Should the preprocessing pipeline (and its inverse) fully preserve the input information, the input label and the processed label should be identical. Any difference represents degradation. The bottom row of Fig. 5 shows exemplar results when applying the previously presented whole volume augmentation pipeline forward and backward to an input label image using nearest neighbour interpolation. Table 2 shows the Dice similarity coefficients for each non-background label class between the input image and the resulting transformed images using traditional and lazy resampling approaches. All results are averaged over all labelled volumes in the dataset. The results for the traditional approach show a significant degradation in label quality with mean Dice similarity coefficients ranging from 0.520 to 0.694 for the foreground classes. The lazy approach has mean Dice similarity coefficients ranging from 0.819 to 0.911 for the foreground classes. While some degradation of the Dice similarity coefficients is inevitable due to the presence of nearest-neighbour resampling of the labels, the effect is mitigated even for challenging and heterogeneous labels such as the tumour core and enhancing tumour labels of Glioma.

**Table 2:** Label round-trip comparison for segmentation labels from the Medical Segmentation Decathlon Brain Tumour task. Dice similarity coefficient between origin and round-tripped samples using traditional and lazy resampling.

Pipeline	Dice similarity coefficients		
	Whole tumour	Tumour core	Enhancing tumour
Traditional	0.694±0.1297	0.520±0.2083	0.598±0.2043
Lazy	0.911±0.0430	0.819±0.1054	0.871±0.1132

### 3.3 Effect of lazy-resampling on semantic segmentation tasks

The previous experiments show the beneficial effects of Lazy Resampling in isolation. It is understood in the literature that adding noise to training data can be beneficial from a generalisation standpoint [11]. As such, it must still be shown that the kind of noise caused by multiple resampling steps in a non-lazy pipeline is biased and thus not useful from a generalisation perspective. This is shown empirically in the following experiment.

We train two models, one on the Medical Segmentation Decathlon Brain Tumour task and one on the Medical Segmentation Decathlon Hippocampus task. For each test, we separate the dataset into five folds. For the Brain Tumour task, we have five folds of 75 labelled volumes per fold. For the Hippocampus task, we have five folds of 52 volumes per fold.

The network is initialised for each run with a set of starting weights and biases which are reproducibly generated by setting a master random number generator seed. The preprocessing pipeline is similarly constructed by setting a master random number generator seed that fixes the random number generator for the ordering of data samples and the application of randomised transforms. The resulting model is then trained with identical learning schedules; both sampling order and random augmentations occur consistently for each iteration for both the non-lazy and lazy pipelines. As such, the only source of difference comes from the pixels output from the pipelines (and potentially any non-determinism within the GPU’s calculations).

Training instability is a common occurrence when training neural networks. Unlike typical training scenarios where randomisation is not fixed, a run with a given seed on a given fold of the dataset will always produce the same result. As such we train both traditional and lazy pipelines and until we have 25 successful training runs for both traditional and lazy pipelines for a given seed and fold. Note also that the same random number seeds are used for both tasks, in numerical order, until sufficient stable training runs have been performed.

The network is a residual UNet architecture [12], [13]. It uses wide residual blocks [14] with a dropout of 0.05 and uses silu [15] for its activations.

#### 3.3.1 Brain tumour task

For the Medical Segmentation Decathlon Brain Tumour task, the network has 5 resolutions going from images of 192 × 192 × 72 voxels down to 12 × 12 × 9 voxels, with the initial downsample being in-plane only. Upsamples are handled by concatenation with the skip connection. The encoder has 1, 2, 2, 2, and 4 blocks respectively in each resolution and the decoder has 1 block per resolution.

The Adam optimiser [16] is used with Dice loss, and the learning rate set to 1e−4.2 with an initial warmup rate of 1e−5 and a reduction of the learning rate every 8 epochs subsequently. The network is trained for approximately 135 epochs of 300 iterations each with a batch size of 2. An Nvidia V100 GPU is used for each run. The training data is preprocessed using the aforementioned whole volume pipeline that consists of the following transforms: spacing, flip, rotate90, zoom and rotate.

#### 3.3.2 Hippocampus task

For the Medical Segmentation Decathlon Hippocampus task, the network has 4 resolutions going from images of 40 × 56 × 40 voxels down to 5 × 7 × 5 voxels. Upsamples are handled by concatenation with the skip connection. The encoder has 1, 2, 2, and 4 blocks respectively in each resolution and the decoder has one block per resolution.

The Adam optimiser [16] is used with Dice loss, and the learning rate set to 1e−4 with an reduction of the learning rate every 50 epochs after the first 100 epochs down to 1e−5. The network is trained for 600 epochs of 300 iterations each with a batch size of 10. An Nvidia V100 GPU is used for each run. The training data is preprocessed using a modified whole volume pipeline that drops the rotate90 operation given the different pixels counts for x and y dimensions and thus consists of the following transforms: resize, flip, zoom and rotate.

**Fig. 6:**
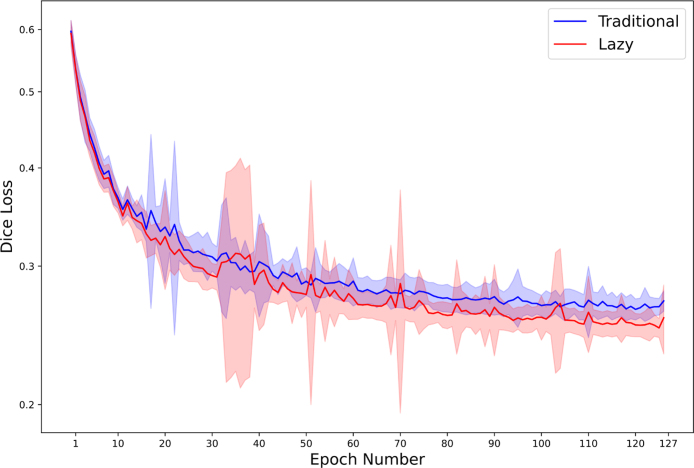
Validation loss (Dice) when training a network for the Brain Tumour semantic segmentation task using a traditionally implemented whole-volume augmentation pipeline and the proposed lazy-approach. The blue and red lines indicate the mean of the Dice loss of 25 runs at each given epoch, for a traditional pipeline and the corresponding lazy pipeline respectively. The blue and red shaded areas represent the standard deviation of the Dice loss of 25 runs at each given epoch, for a traditional pipeline and the corresponding lazy pipeline respectively.

**Fig. 7:**
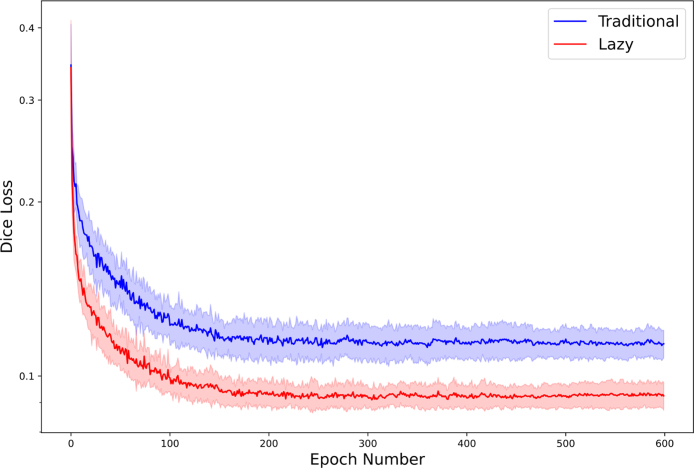
Validation loss (Dice) when training a network for the Hippocampus semantic segmentation task using a traditionally implemented whole volume augmentation pipeline and the proposed lazy-approach. The blue and red lines indicate the mean of the Dice loss of 25 runs at each given epoch, for a traditional pipeline and the corresponding lazy pipeline respectively. The blue and red shaded areas represent the standard deviation of the Dice loss of 25 runs at each given epoch, for a traditional pipeline and the corresponding lazy pipeline respectively.

The mean Dice similarity coefficient results are shown in Table 3. The lazy pipeline achieves a 0.0122 Dice loss improvement (4.9%) for the Brain Tumour task and a 0.0181 Dice loss improvement (20.9%) for the Hippocampus task over the traditional processing pipeline across 25 pairs of runs that each have exactly the same starting conditions and randomisation. The dependent T Test for paired samples has been performed on the best scores for each corresponding traditional and lazy run and indicates a statistically significant improvement (p-value of <1e−9) for both the Brain Tumour task and the Hippocampus task. Fig. 6, Fig. 7 show the means and standard deviations of per-epoch losses for the Brain Tumour and Hippocampus segmentation tasks respectively.

**Table 3:** Mean and standard deviation of validation Dice Similarity Coefficients (DSC) over 25 training runs of semantic segmentation with the whole volume pipeline using traditional and lazy resampling respectively.

Pipeline	Brain Tumour DSC		Hippocampus DSC	
	DSC (mean)	DSC (var)	DSC (mean)	DSC (var)
Traditional	0.7400	6.066e−05	0.8954	2.515e−05
Lazy	0.7522	6.154e−05	0.9135	6.106e−06

## 4 Discussion

The results demonstrate that Lazy Resampling is able to better preserve information compared to traditional preprocessing pipelines when carrying out multiple spatial transformations on images and semantic labels. This is highlighted both in the forward pass experiment and the round trip experiment. In particular, the round trip experiment highlights the divergence between artifacts generated by successive linear resamples and those generated for the labels by successive nearest neighbour resamples. Reducing the number of resamples helps minimise this effect and minimise the misalignment of resampled labels relative to resampled data.

Lazy Resampling can also accelerate preprocessing pipelines as it can carry out fewer CPU and memory-intensive resampling operations. This is demonstrated through analysis of the pipelines operating in isolation and through training of a UNet on the Medical Segmentation Decathlon Brain Tumour dataset, which results in faster convergence rates than traditional preprocessing with all other factors fixed.

## 5 Conclusion

We have presented Lazy Resampling; a method that enables deep learning researchers to build pipelines that take advantage of computer graphics best practice by compositing together transform descriptions and applying them as a single resampling operation where possible.

We have demonstrated that Lazy Resampling mitigates information loss through the avoidance of lossy resampling steps and otherwise destructive operations such as crops and rotates without having to add complexity to a pipeline in the form of pre-emptive padding of the data.

We have further demonstrated that, for semantic segmentation labels, Lazy Resampling is able to mitigate catastrophic degradation that comes with repeated nearest-neighbour resampling steps, particularly when round-tripping labels, making otherwise impractical workflows practical.

Finally, we have demonstrated statistically significant benefits on semantic segmentation training tasks, showing improved performance of lazy pipelines over their corresponding traditional counterparts from identical starting conditions and with identical randomness applied during training.

### 5.1 Future work

There is an active roadmap for Lazy Resampling development. Amongst the upcoming features for Lazy Resampling are:

• *Lazy execution for transforms that access data.* Certain transforms, such as those that ensure patches contain all semantic segmentation classes, need pending transforms to be executed so that they can perform their calculations correctly. An intermediate sample at this point in the pipeline may not be necessary or desirable, however. Such transforms can perform the resample for their own analysis, but then discard the resampled data and preserve the pending transforms for the full pipeline execution.
• *General matrix/grid operations.* At present, grid-based transforms cause resampling to occur. This is a limitation of the current implementation, but there is no technical reason why grid and matrix transforms cannot be lazily chained. In the case of a grid followed by a matrix, the matrix transforms each vector in the grid. Grid/grid transformations are more involved and require a change in the way grids are represented.
• *Lazy noise calculation.* At present, noise transforms are not lazy and interleaving them with spatial transforms results in intermediate resamples. This is not necessary, as noise can be calculated and applied based on the partial composed transform at which the noise is defined and the composed transform at which the noise is applied. This has the effect of transforming the noise as if it had been introduced at the appropriate point in the pipeline.

### 5.2 Code availability

The work presented here and the code required to reproduce the experiments are available for download at https://github.com/KCL-BMEIS/LazyResampling. A partial implementation is integrated into the popular open-source MONAI library as of version 1.2, available at https://monai.io and is under active development.

## CRediT authorship contribution statement

**Benjamin Murray:** Conceptualization, Data curation, Formal analysis, Investigation, Methodology, Software, Validation, Visualization, Writing – original draft, Writing – review & editing. **Richard Brown:** Methodology, Software, Writing – review & editing. **Pengcheng Ma:** Software, Writing – review & editing. **Eric Kerfoot:** Software, Writing – review & editing. **Daguang Xu:** Writing – review & editing. **Andrew Feng:** Writing – review & editing. **Jorge Cardoso:** Funding acquisition, Project administration, Supervision. **Sebastien Ourselin:** Funding acquisition, Methodology, Project administration, Supervision, Validation, Writing – review & editing. **Marc Modat:** Formal analysis, Funding acquisition, Investigation, Methodology, Project administration, Supervision, Validation, Writing – original draft, Writing – review & editing.

## Declaration of competing interest

The authors declare that they have no known competing financial interests or personal relationships that could have appeared to influence the work reported in this article.

## Footnotes

1 https://pytorch.org/vision.

2 https://github.com/MIC-DKFZ/batchgenerators.

3 https://github.com/PhoenixDL/rising.
